# Heterogeneity in Effects of Automated Results Feedback After Online Depression Screening: Secondary Machine-Learning Based Analysis of the DISCOVER Trial

**DOI:** 10.2196/70001

**Published:** 2025-08-21

**Authors:** Matthias Klee, Byron C Jaeger, Franziska Sikorski, Bernd Löwe, Sebastian Kohlmann

**Affiliations:** 1 Department of General Internal Medicine and Psychosomatics, Heidelberg University, Im Neuenheimer Feld 410, Heidelberg, 69120, Germany, 49 62215632879; 2 Department of Biostatistics and Data Science, Wake Forest University School of Medicine, Winston-Salem, NC, United States; 3 Department of Psychosomatic Medicine & Psychotherapy, University Medical Center Hamburg-Eppendorf, Hamburg, Germany

**Keywords:** heterogeneity of treatment effects, treatment heterogeneity, causal random forest, random forest, depression, depression screening, DISCOVER, feedback

## Abstract

**Background:**

Online depression screening tools may increase uptake of evidence-based care and consequently lead to symptom reduction. However, results of the DISCOVER trial suggested no effect of automated results feedback compared with no feedback after online depression screening on depressive symptom reduction six months after screening. Interpersonal variation in symptom representation, health care needs, and treatment preferences may nonetheless have led to differential response to feedback mode on an individual level.

**Objective:**

The aim of this study was to examine heterogeneity of treatment effects (HTE), that is, differential responses to two feedback modes (tailored or nontailored) versus no feedback (control) following online depression screening.

**Methods:**

We used causal forests, a machine learning method that applies recursive partitioning to estimate conditional average treatment effects (CATEs). In this secondary data analysis of the DISCOVER trial, eligible participants screened positive for at least moderate depression severity but had not been diagnosed or treated for depression in the preceding year. The primary outcome was heterogeneity in depression severity change, over a and six-month follow up period, measured with the Patient Health Questionnaire-9. Analysis comprised exploration of average treatment effects (ATE), HTE, operationalized with the area under the targeting operator characteristic curve (AUTOC), and differences in ATE when allocating feedback based on predicted CATE. We extracted top predictors of depression severity change, given feedback and explored high-CATE covariate profiles. Prior to analysis, data was split into training and test sets (1:1) to minimize the risk of overfitting and evaluate predictions in held-out test data.

**Results:**

Data from 946 participants of the DISCOVER trial without missing data were analyzed. We did not detect HTE; no versus nontailored feedback, AUTOC −0.48 (95% CI −1.62 to 0.67, *P=*.41); no versus tailored feedback, AUTOC 0.06 (95% CI −1.21 to 1.33, *P=*.93); and no versus any feedback, AUTOC −0.20 (95% CI −1.30 to 0.89, *P=*.72). There was no evidence of alteration to the ATE in the test set when allocating feedback (tailored or nontailored) based on the predicted CATE. By examining covariate profiles, we observed a potentially detrimental role of control beliefs, given feedback compared with no feedback.

**Conclusions:**

We applied causal forests to describe higher-level interactions among a broad range of predictors to detect HTE. In absence of evidence for HTE, treatment prioritization based on trained models did not improve ATEs. We did not find evidence of harm or benefit from providing tailored or nontailored feedback after online depression screening regarding depression severity change after six months. Future studies may test whether screening alone prompts behavioral activation and downstream depression severity reduction, considering the observed uniform changes across groups.

## Introduction

Online depression screening may foster early identification of individuals with depressive symptoms and reinforce help-seeking behavior [1,2]. However, recently published results of the DISCOVER trial (ClinicalTrials.gov, NCT04633096) suggested no effect of automated results feedback compared with no feedback after online depression screening on depressive symptom reduction six months after screening [1].

Randomized controlled trials (RCT) are the gold-standard method for evaluating intervention efficacy. Still, evidence-based medicine incorporates the notion of differential responses to interventions on a person-level, potentially masking harms or benefits at the group-level [3]. Thus, it is important to examine individual-level responses to feedback.

Machine-learning (ML) methods have previously been adapted to examine such heterogeneity of treatment effects (HTE) [4-6]. ML can circumvent the issue of multiple testing through cross-validation and by design, account for higher level interactions [7]. ML approaches to estimate HTE have been successfully applied in health care literature, especially in cardiology and psychiatry [5,8].

The aim of this paper was to investigate the presence of HTE in response to feedback (no feedback ie, control group vs nontailored or tailored, ie, intervention), following online depression screening. We tested for the presence of HTE, based on person-level characteristics at baseline, with heterogeneity in depression severity change at six months as the primary outcome. The efficacy of allocating feedback to individuals with more favorable predicted conditional treatment response was examined in exploratory analysis.

## Methods

### Study Sample and Design

DISCOVER is a three-armed RCT examining change in depression severity six months after online screening with tailored, nontailored, or no feedback (control). The study was advertised as a study on stress and psychological well-being [1]. Recruitment strategies involved print and online advertisements on social media platforms and in a nationwide online access survey panel in Germany [1]. Eligible participants were 18 years or older, proficient in German, and screened positive for at least moderate depression severity (Patient Health Questionnaire-9, PHQ-9 ≥10) [1]. Participants with missing data, or those with a diagnosis of or treatment for depression in the previous year were excluded.

Feedback comprised depression screening results, a recommendation to consult a mental health care professional or general practitioner, and further information regarding depression and related treatment based on national guidelines [1,9]. For the tailored feedback group, feedback was adapted according to participants’ symptom profiles, treatment preferences, and available guideline-recommended options [1,9].

### Ethical Considerations

Review and approval was provided by the Ethics Committee of the Hamburg Medical Chamber (PV7039) [9]. Participants provided online informed consent covering secondary data analyses [1]. Participants received a € 5 (US $5.85) voucher for compensation upon each completed follow-up. Data was deidentified prior to analysis.

### Main Outcomes and Measures

The primary outcome was heterogeneity in depression severity change six months after online screening. Depression severity was measured with the PHQ-9 [10]. Predictors involved baseline depression (PHQ-9), anxiety (Generalized Anxiety Disorder Scale-7; GAD-7 [11]), and somatic symptom severity (Somatic Symptom Scale-8; SSS-8 [12]), health-related quality of life (EuroQoL-5 Dimension-5 Level visual analogue scale [EQ-5D-5 L VAS]) [13], illness beliefs (Brief Illness Perception Questionnaire; B-IPQ [14,15]), patient history, depression-related risk factors, and sociodemographic characteristics (Table S1 in Multimedia Appendix 1).

### Statistical Analysis

Causal Forests (CF) [5,6] have previously been used to investigate HTE [8] (Box S1 in Multimedia Appendix 1). CFs estimate conditional average treatment effects (CATE), which approximate individual-level treatment effects (ITE). ITE cannot be inferred directly since only one potential outcome is realized per participant. Thus, CATE are more granular than average treatment effects (ATE) but less granular than ITE.

Two CFs were trained based on either training (tau-forest) or test data (eval-forest), with a random split (1:1). CFs were trained with 2000 trees, a sample fraction of 0.5, a minimum node size of 5, and mtry = 30. ATE and CATE were computed by contrasting intervention (nontailored [1], tailored [2], or any [1/2] feedback) to no feedback (control) with depression severity change as the outcome and a propensity score for treatment allocation (*P=*.50) [4,5].

To assess the presence of HTE discretely, tau-forest predictions of CATE for the test data were grouped into quartiles. Then, ATE was estimated in each quartile using the eval-forest. To assess the presence of HTE continuously, we computed the area under the targeting operator characteristic curve (AUTOC) and tested for the presence of HTE with a significance test for AUTOC (H_1_:AUTOC≠0) [4]. Significance of AUTOC was tested two-sided, with bootstrapped standard errors (n=200 bootstrap replicates).

For a comprehensive overview of model evaluation and sample code, see Box S2 in Multimedia Appendix 1, Sverdrup, Petukhova [4] and Klee [16]. Analyses were conducted using R (version 4.3.1; R Foundation for Statistical Computing) using the *grf* package [17].

## Results

### Baseline Characteristics

After visual inspection of missingness patterns, 19 participants were removed, assuming missingness at random (Table S2 in Multimedia Appendix 1). In total, 946 participants were eligible for analysis. Participants were aged 18 to 79 years, mean 37.20 (SD 13.98) (Table 1).

**Table 1.** Baseline characteristics of participants in the analytic data set (N=946).

Characteristic	No feedback (n=318)	Nontailored feedback (n=313)	*P* value*^a^*	Tailored feedback (n=315)	*P* value*^a^*
Age in years, mean (SD)	36.4 (13.7)	38.2 (13.8)	.11	37.0 (14.4)	.62
Gender, n (%)	.85^b^	.73^b^
Women	232 (73.0)	222 (70.9)	—	221 (70.2)	—
Men	83 (26.1)	88 (28.1)	—	91 (28.9)	—
Other	3 (0.9)	3 (1.0)	—	3 (1.0)	—
Education, n (%)	.66	.55
<10 years	55 (17.3)	59 (18.8)	—	47 (14.9)	—
≥10 years	94 (29.6)	99 (31.6)	—	104 (33.0)	—
University entrance qualification	169 (53.1)	155 (49.5)	—	164 (52.1)	—
Depression severity (PHQ-9^c^), mean (SD)	14.8 (4.03)	14.7 (4.09)	.78	14.8 (3.82)	.94
Somatic symptom severity (SSS-8^d^), mean (SD)	14.6 (5.23)	14.5 (5.13)	.90	14.3 (5.32)	.57
Anxiety severity (GAD-7^e^), mean (SD)	12.0 (4.32)	12.5 (4.23)	.19	12.0 (4.29)	.94

a *P* values for pairwise comparisons with the ‘no feedback’ group. Continuous characteristics were compared with Student’s *t*-test, categorical characteristics were compared with *χ*2 tests.

b *χ*2 approximation may be incorrect due to small cell size. Analysis based on men and women only replicated findings (*P=*.63 for nontailored feedback vs no feedback, *P=*.48 for tailored feedback vs no feedback).

c PHQ-9=Patient Health Questionnaire-9.

d SSS-8=Somatic Symptom Scale-8.

e GAD-7=Generalized Anxiety Disorder Scale-7.

### Average Treatment Effect

We did not find evidence of non-zero ATE in either tau- or eval-forests, suggesting no benefit of providing any form of feedback compared with the control (no feedback) (Table 2).

**Table 2.** Average treatment effects for pairwise comparison of feedback conditions.

Comparison	Tau-forest^a^	Eval-forest^b^
	ATE^c^ (SE)	ATE (SE)
No feedback versus nontailored feedback	0.04 (0.54)	−0.24 (0.55)
No feedback versus tailored feedback	−0.38 (0.57)	−0.16 (0.57)
No feedback versus any feedback	0.07 (0.48)	−0.48 (0.49)

a Tau-forest is based on training data.

b Eval-forest is based on test data.

c ATE: Average treatment effect.

### Heterogeneity in Treatment Effects

**Figure 1.**
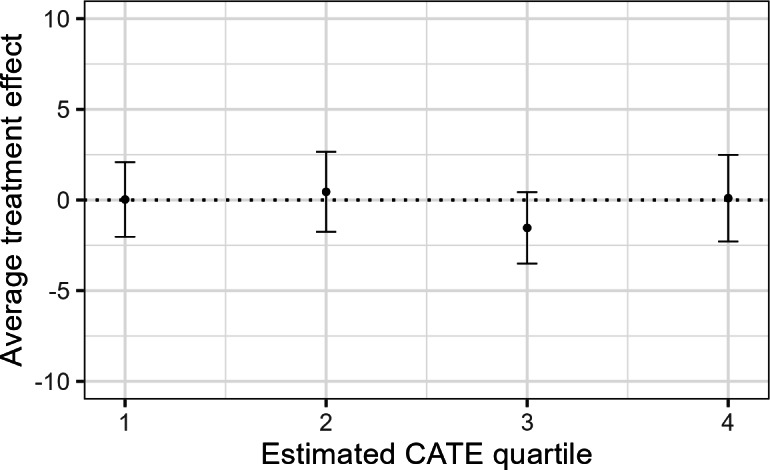
Average treatment effects in participant groups reflecting quartiles of predicted CATE from lowest (L) to highest (R). CATE was predicted in test data with the tau-forest. ATE was estimated within quartiles with the evaluation forest (based on test data). Positive values indicate less favorable ATE. ATE: average treatment effect; CATE: conditional average treatment effect.

There was no evidence of HTE when comparing nontailored (Figure 1) or any feedback with control (Figure S4 in Multimedia Appendix 1). However, there was a lower (ie, more favorable) ATE when comparing tailored feedback with control among participants with predicted CATE in the second most favorable quartile regarding depression severity change (Figure S1 in Multimedia Appendix 1).

**Figure 2.**
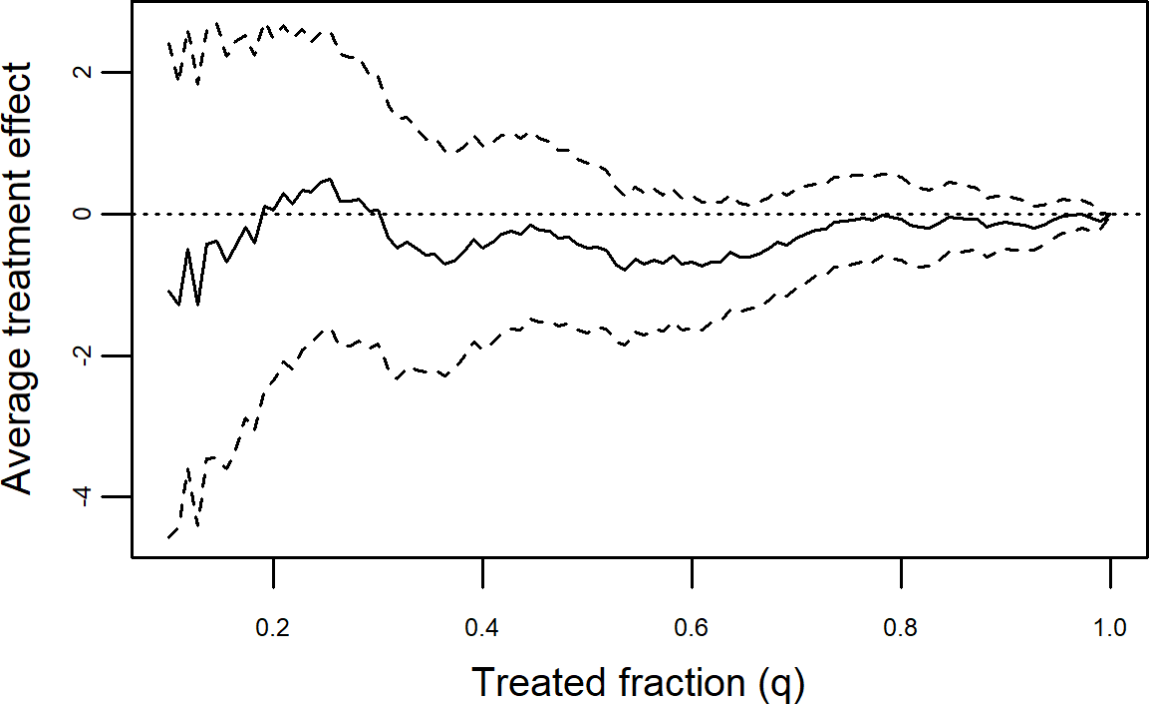
Targeting operator characteristic curve plot. The dashed lines are pointwise 95% confidence intervals conditional on the estimated CATE function, (ie, the tau-forest based on the training data). The y-lab illustrates the benefit of providing feedback only to a fraction of participants based on their CATE (ie, treatment priority score), over treating everyone (difference in average treatment effects; ie, PHQ-9 change six months after screening). The x lab illustrates the fraction treated from highest (L) to lowest (R) CATE. Positive values indicate less favorable ATE. ATE: average treatment effect; CATE: conditional average treatment effect.

AUTOC estimates (Figures S2 and S5 in Multimedia Appendix 1) did not suggest the presence of HTE in any comparison: AUTOC −0.48 95% CI −1.62 to 0.67, *P=*.41, nontailored; AUTOC 0.06, 95% CI −1.21 to 1.33, *P=*.93 tailored; AUTOC −0.20 (95% CI −1.30 to 0.89, *P=*.72 any feedback vs control). Allocating feedback based on predicted CATE did not substantially alter ATE (Figure 2). This is consistent with the near-zero AUTOC estimates, and indicates limited potential for altering the effects of feedback mode regarding depression severity change through targeted allocation.

### Top Predictors of Harm or Benefit From Treatment

The most important predictors of the tau-forest (comparing nontailored feedback with control) were items assessing depression–related treatment control belief (B-IPQ), trouble relaxing (GAD-7), anxiety severity (GAD-7) and trouble sleeping (SSS-8). For tailored feedback compared with control, items denoting age, somatic symptom (SSS-8) and anxiety (GAD-7) severity and depression-related treatment control belief (B-IPQ) were most important. For any feedback compared with control, illness beliefs (B-IPQ) were most important: treatment and personal control, concern, and emotional response.

### Sensitivity Analyses

Higher treatment control (nontailored feedback vs control) and personal control beliefs (any feedback vs control) were the only predictors significantly associated with a linear approximation of CATE (Table 3 and Tables S3 and S4 in Multimedia Appendix 1). Both predictors were associated with less favorable CATE estimates, suggesting less favorable depression severity change at follow-up, given feedback.

**Table 3.** Best linear projection for top predictors of the causal forest with training data.

Term	Estimate	SE	*P* value
GAD-7^a^ Item 4: How frequent did you feel impaired by the following symptoms during the past 2 weeks? – Trouble relaxing (0 not at all to 3 almost every day)	1.60	0.92	.08
Illness Perception Item 4: How much do you think a treatment can help with these complaints? (0 not at all to 10 extremely helpful)	0.47	0.21	.03
GAD-7^a^ Sum score (0 to 21)	0.00	0.17	.99
SSS-8^b^ Item 8: How strongly did you feel impaired by the following complaints during the past 7 days? – Trouble sleeping? (0 not at all to 4 very strongly)	0.61	0.50	.23

a GAD-7: Generalized Anxiety Disorder Scale-7.

b SSS-8: Somatic Symptom Scale-8.

**Figure 3.**
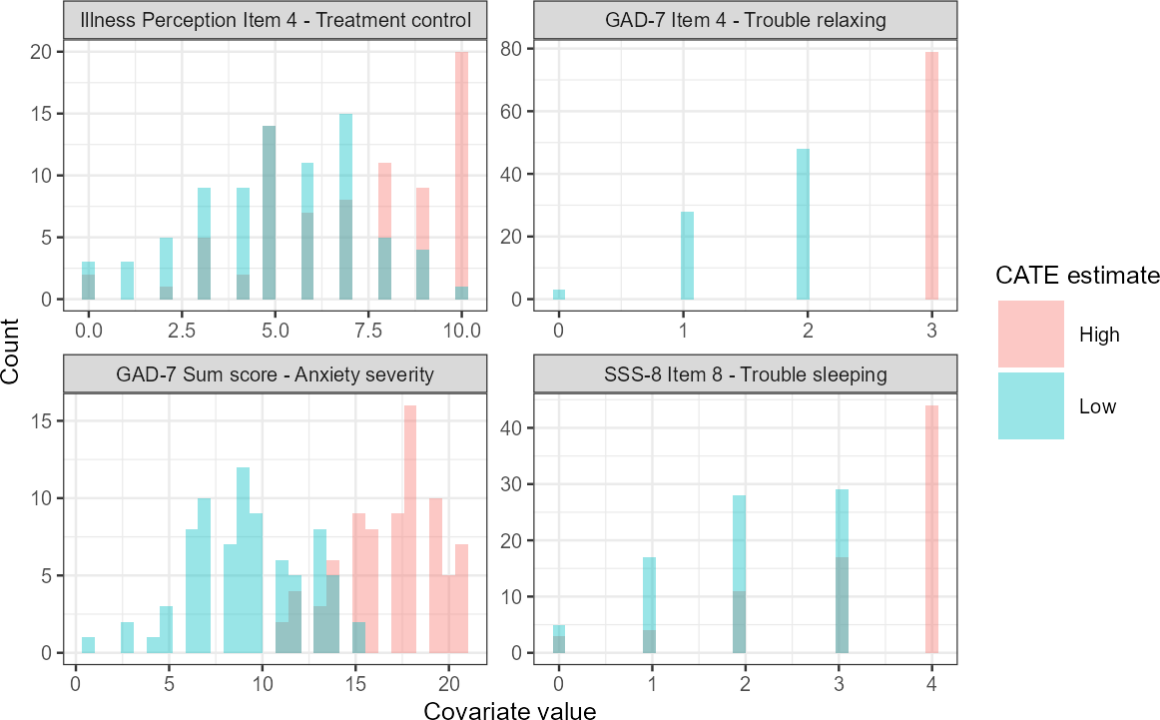
Covariate profiles for test data with high (upper 25%, magenta) or low (lower 25%, cyan) predicted CATE based on tau-forest. CATE: conditional average treatment effect; GAD-7: Generalized Anxiety Disorder Scale-7; SSS-8: Somatic Symptom Scale-8.

Covariate profiles of the most important predictors are depicted in Figure 3 and Figures S3 and S6 in Multimedia Appendix 1. Overall, a higher treatment control belief was more frequent in the highest (least favorable) CATE quartile. Findings are less clear for remaining top four most important predictors.

Examining model calibration, CATE functions estimated in tau-forests did not significantly contribute to predicting change in depression severity at follow-up above group-level mean prediction (Table S5 in Multimedia Appendix 1).

## Discussion

### Principal Results

By applying CF to the DISCOVER online RCT, we did not find evidence for HTE with feedback (tailored, nontailored, or any) regarding change in depression severity six months after screening. As such, no apparent subgroup with an altered response to any type of feedback mode was detected.

### Limitations

First, the selection of participants with at least moderate depression severity increases the likelihood of regression to the mean, which may have impeded the investigation of HTE. Second, generalizability of findings is limited to individuals participating in an online study about psychological well-being, who may exhibit distinct severity trajectories. Third, follow-up time may have been too short to detect variation in severity change, given, for example, the latency of help seeking.

### Comparison With Prior Work

Our findings are in line with previous results showing no significant average benefit of any feedback mode for change in depression severity [1]. Beyond average effects, we investigated within-group harms and benefits in accordance with an evidence-based medicine approach [3]. We complement previous research suggesting no between-group detriments [18] with results suggesting that there are no latent subgroups that vary in their response to feedback. In contrast to previous research, our findings are valid irrespective of a priori defined categorical operationalizations of harmful or beneficial events. We show that included predictors do not alter response to feedback, providing an approximated assessment of individual-level harms and benefits [1,18].

Critically, sensitivity analyses suggested limited calibration of trained models. However, when testing HTE with CF, accurate mean prediction of the primary outcome is a first step necessary to detect deviations from it (ie, HTE). Previous research illustrates the notorious difficulty of predicting future depression courses, even with updated analytic tools [19-21]. Still, by employing a nonparametric method, we provide HTE estimates, that can account for a broad range of heterogeneity mechanisms potentially underlying depressive symptom trajectories and their variation following automated results feedback [8].

### Conclusions

Applying CF, we could examine a broad range of predictors to detect HTE. In the absence of evidence for HTE, treatment prioritization based on trained models did not improve ATEs. We did not find evidence for harm or benefit of providing feedback after online depression screening regarding depression severity change after six months. Future studies may test if screening alone prompts behavioral activation and downstream depression severity reduction, considering the observed uniform changes across groups [22].

## Supplementary material

10.2196/70001 Multimedia Appendix 1 Supplementary material containing a detailed list of included predictors, descriptive characteristics of participants with or without missing data, results for comparisons of no feedback with tailored or any feedback, sensitivity analyses of calibration and more detailed description of causal forests to detect heterogeneity of treatment effects, and the model evaluation strategy.

## Abbreviations

CF: Causal Forests
ATE: Average treatment effects
AUTOC: Area under the targeting operator characteristic curve
B-IPQ: Brief Illness Perception Questionnaire
CATE: conditional average treatment effects
GAD-7: Generalized Anxiety Disorder Scale-7
HTE: Heterogeneity of treatment effects
ITE: Individual level treatment effects
ML: Machine learning
PHQ-9: Patient Health Questionnaire-9
RCT: Randomized controlled trial
SSS-8: Somatic Symptom Scale-8
TOC: Targeting operator characteristic (curve)
