# Supplementary material for: Heterogeneity in Effects of Automated Results Feedback After Online Depression Screening: Secondary Machine-Learning Based Analysis of the DISCOVER Trial
Source: JMIR AI. 2025 Aug 21;4:e70001. doi: 10.2196/70001 (PMC12375799; doi:10.2196/70001)
Supplement: Multimedia Appendix 1 [file ai-v4-e70001-s001.docx]

## Supplementary Material

### Predictors

Table S1. Complete list of predictors used to model Causal Forests.

| **Variable list** | **Label** | **Response levels** | **Used** |
| --- | --- | --- | --- |
| **trial_id** | Running trial ID |  | no |
| **condition** | Study arm | Tailored Feedback  Non-tailored Feedback  No Feedback (control) | yes |
| **t0_t3_change_ phq9** | PHQ-9 change baseline – six-month follow-up | Numeric (Range -27 to 17) | yes |
| **Patient Health Questionnaire-9** | How frequent did you feel impaired by the following complaints during the past 2 weeks. | Numeric (Range 0 Not at all to 4 Nearly every day) |  |
| **t0_phq9_1** | Little interest or pleasure in doing things. |  | yes |
| **t0_phq9_2** | Feeling down, depressed, or hopeless. |  | yes |
| **t0_phq9_3** | Trouble falling or staying asleep or sleeping too much. |  | yes |
| **t0_phq9_4** | Feeling tired or having little energy. |  | yes |
| **t0_phq9_5** | Poor appetite or overeating. |  | yes |
| **t0_phq9_6** | Feeling bad about yourself or that you are a failure or have let yourself or your family down. |  | yes |
| **t0_phq9_7** | Trouble concentrating on things, such as reading the newspaper or watching television. |  | yes |
| **t0_phq9_8** | Moving or speaking so slowly that other people could have noticed. Or the opposite being so fidgety or restless that you have been moving around a lot more than usual. |  | yes |
| **t0_phq9_9** | Thoughts that you would be better off dead, or of  hurting yourself. |  | yes |
| **t0_phq9_sum** | Total PHQ-9 score at baseline | Numeric (Range 10 to 27) | yes |
| **severity** | Stratum (severity of depression) | Moderate: PHQ-9 10 – 14 Severe: PHQ-9 >= 15 | yes |
| **t0_pref_1** | If you suspected that you were suffering from depression, which type of healthcare provider would you consult first? | General practitioner  Psychotherapist  I don’t know | yes |
| **t0_vordia_0** | Have you ever been diagnosed with depression or burnout? | Never  In the last year (excluded)  In the last five years  In the last ten years  More than ten years ago | yes |
| **Depression risk factors** |  | Yes  No |  |
| **t0_deprisk_1** | Are you suffering from anxiety? |  | yes |
| **t0_deprisk_2** | Are you suffering from an addiction (drugs, computer game addiction, gambling addiction, etc.)? |  | yes |
| **t0_deprisk_3** | Have you had a life event in the past that still burdens you today? |  | yes |
| **t0_deprisk_4** | Have you been suffering from persistent physical complaints for at least six months (e.g., back pain, headaches, nausea)? |  | yes |
| **t0_deprisk_5** | Have you suffered from mood swings or a depressed mood in the last month? |  | yes |
| **t0_deprisk_6** | Do you have any chronic physical illnesses (e.g., heart disease, diabetes, asthma, etc.)? Based on t0_deprisk_7_0 to t0_deprisk_7_8 |  | yes |
| **t0_deprisk_7_0** | Heart disease |  | no |
| **t0_deprisk_7_1** | Diabetes |  | no |
| **t0_deprisk_7_2** | Respiratory diseases (e.g., asthma, COPD, etc.) |  | no |
| **t0_deprisk_7_3** | Gastrointestinal diseases |  | no |
| **t0_deprisk_7_4** | Neurological or nerve disorders (e.g., multiple sclerosis, stroke, etc.) |  | no |
| **t0_deprisk_7_5** | Pain disorders |  | no |
| **t0_deprisk_7_6** | Cancer |  | no |
| **t0_deprisk_7_7** | Rheumatological diseases and/or joint diseases |  | no |
| **t0_deprisk_7_8** | other illness(es) |  | no |
| **t0_deprisk_8** | Do you feel supported by your social environment? |  | yes |
| **t0_deprisk_9** | Have you been diagnosed with any other mental disorder? | Never  In the last year  In the last five years  In the last ten years  More than ten years ago | yes |
| **t0_deprisk_10** | Do you have family members who suffer from mental health issues (e.g., depression, anxiety, eating disorders, substance abuse)? |  | yes |
| **t0_deprisk_11** | Are there known cases of suicides or suicide attempts in your family history? |  | yes |
| **t0_deprisk_12** | Are you currently trying to get pregnant? |  | no |
| **t0_deprisk_13** | Are you currently pregnant? |  | no |
| **t0_deprisk_14** | Have you given birth in the last 6 months? |  | no |
| **t0_deprisk_15** | Are you currently breastfeeding? |  | no |
| **t0_deprisk_16** | Do you suffer from premenstrual syndrome (PMS)? |  | no |
| **t0_deprisk_17** | Are you in menopause? |  | no |
| **t0_rfs_sum** | Risk Factor Score - Sum of reported risk factors (t0_deprisk_1 to t0_deprisk_6, t0_deprisk_8 (inverted), t0_deprisk_9 to t0_deprisk_17) | Numeric (Range 0 to 16) | yes |
| **t0_sms_sum** | Somatic Morbidity Score - Sum of the reported somatic illnesses (heart disease, respiratory diseases, diabetes, intestinal disease, neurological disease, pain disorder, cancer, rheumatological disease, other) | Numeric (Range 0 to 9) | no |
|  |  |  |  |
| **European Quality of Life 5 Dimensions 5 Level Version** | Please chose the statement that fits your health status today best | I have no problems …  I have a little bit of a problem …  I have some problems …  I have a lot of problems …  I cannot … |  |
| **t0_eq5d_1** | Mobility | … walking about | yes |
| **t0_eq5d_2** | Looking after oneself | … washing or dressing myself | yes |
| **t0_eq5d_3** | Usual activities (for example, going to school, hobbies, sports, playing, doing things with family or friends) | … doing my usual activities | yes |
| **t0_eq5d_4** | Pain or physical complaints | … pain or complaints | yes |
| **t0_eq5d_5** | Anxiety / low mood | I am a bit anxious or depressed  I am quite anxious or depressed  I am really anxious or depressed  I am extremely anxious or depressed | yes |
| **t0_eq5d_6** | If you imagine a scale from 0 to 100, with 0 representing the worst possible health state and 100 representing the best, where would you place yourself? | Numeric (Range 0 to 100) | yes |
| **t0_demo_1** | How big is the city you live in? | < 4,999 residents  5,000 to 19,999 residents  20,000 to 99,999 residents  >= 100,000 residents | yes |
| **t0_demo_2_eastern_state** | Residency in Brandenburg, Mecklenburg-Western Pomerania, Saxony, Saxony-Anhalt or Thuringia | Yes  No | yes |
| **t0_demo_2_city_state** | Residency in Berlin, Bremen, Hamburg | Yes  No | yes |
| **t0_demo_3** | How are you insured? | Statutory  Private | yes |
| **t0_demo_9** | Would you consider yourself a migrant or a person with a migration background? | Yes  No | yes |
| **t0_demo_10** | Do you think others here in Germany would consider you a migrant or a person with a migration background? | Yes  No | yes |
| **t0_demo_11** | What is your gender? | Diverse  Female  Male | yes |
| **t0_demo_12** | How old are you? | Numeric (Range 18 to 79 years) | yes |
| **t0_demo_13** | What is your marital status? | Married/in a relationship  Single, divorced, widowed, other | yes |
| **t0_demo_14** | What is your living situation? | Living together with others  Living alone | yes |
| **t0_demo_15_ mod** | Education level in categories | Low (< 10 years of education)  Middle (>= 10 years of education)  High (university entrance qualification) | yes |
| **t0_demo_17** | What is your employment status? | Not working  Retired  Unemployed  Working part-time or by the hour  Employed | yes |
| **t0_nikotin_1** | Do you smoke (cigarettes, e-cigarettes, cigarillos, pipe)?  Response options: | Yes  No | yes |
| **t0_alk_sum** | Alcohol consumption (Alcohol Use Disorders Identification Test; AUDIT-C) total score | Numeric (Range 0 to 12) | yes |
| **Illness Perception** |  |  |  |
| **t0_bipq_1** | How much do these complaints affect your life? | Numeric (Range 0 no impairment to 10 very strong impairment) | yes |
| **t0_bipq_2** | How long do you think these complaints will continue? | Numeric (Range 0 just a short time to 10 forever) | yes |
| **t0_bipq_3** | How much control do you feel you have over your complaints? | Numeric (Range 0 no control at all to 10 full control) | yes |
| **t0_bipq_4** | How much do you think a treatment can help with these complaints? | Numeric (Range 0 not at all to 10 extremely helpful) | yes |
| **t0_bipq_5** | How concerned are you about your complaints? | Numeric (Range 0 not concerned at all to 10 extremely concerned) | yes |
| **t0_bipq_6** | How much do you feel you understand your complaints? | Numeric (Range 0 not at all to 10 very clear) | yes |
| **t0_bipq_7** | How much do these complaints affect you emotionally? (e.g., do they make you angry, scared, upset or depressed?) | Numeric (Range 0 not at all to 10 extremely) | yes |
| **t0_bipq_8** | Is this the first time in your life that you are experiencing such complaints? | No, I know these complaints from earlier phases of my life  Yes, this is the first time | yes |
| **t0_bipq_9** | Can you imagine that you are currently suffering from depression? | No  Yes  Maybe | yes |
| **t0_bipq_10** | In your opinion, what are the causes of these complaints? If applicable, please select one answer for each of the following three areas that you think fits best. | Problems within the family  Problems with friends  Problems with the partner  Loss of an important person  Financial problems or instability  Problems with or stress at work | no |
| **t0_bipq_11** | In your opinion, what are the causes of these complaints? If applicable, please select one answer for each of the following three areas that you think fits best. | My attitude, for example, negative thoughts about life  My usual behavioral patterns  Stressful events of the past  Events from my childhood  Too high expectations of myself | no |
| **t0_bipq_12** | In your opinion, what are the causes of these complaints? If applicable, please select one answer for each of the following three areas that you think fits best. | Physical disease chemical or hormonal causes  Hereditary; caused by genetic predispositions | no |
| **Somatic Symptom Scale-8 (SSS-8)** | How strongly did you feel impaired by the following complaints during the past 7 days? | Numeric (Range 0 not at all to 4 very strongly) |  |
| **t0_sss8_1** | Stomach or bowel problems |  | yes |
| **t0_sss8_2** | Back pain |  | yes |
| **t0_sss8_3** | Pain in your arms, legs, or joints (knees, hips, etc.) |  | yes |
| **t0_sss8_4** | Headaches |  | yes |
| **t0_sss8_5** | Chest pain or shortness of breath |  | yes |
| **t0_sss8_6** | Dizziness |  | yes |
| **t0_sss8_7** | Feeling tired or having low energy |  | yes |
| **t0_sss8_8** | Trouble sleeping |  | yes |
| **t0_sss8_sum** | Total SSS-8 score at baseline | Numeric (Range 0 to 32) | yes |
| **Generalized Anxiety Disorder Scale-7 (GAD-7)** | How frequent did you feel impaired by the following symptoms during the past 2 weeks | Numeric (Range 0 not at all to 3 almost every day) |  |
| **t0_gad7_1** | Feeling nervous, anxious, or on edge |  | yes |
| **t0_gad7_2** | Not being able to stop or control worrying |  | yes |
| **t0_gad7_3** | Worrying too much about different things |  | yes |
| **t0_gad7_4** | Trouble relaxing |  | yes |
| **t0_gad7_5** | Being so restless that it is hard to sit still |  | yes |
| **t0_gad7_6** | Becoming easily annoyed or irritable |  | yes |
| **t0_gad7_7** | Feeling afraid, as if something awful might happen |  | yes |
| **t0_gad7_sum** | Total GAD-7 score at baseline | Numeric (Range 0 to 21) | yes |

*Note*. Categorical predictors with more than two levels were one-hot-encoded prior to analyses. Predictors with missing values in more than 10% of the sample were not included in the primary analyses.

### Missing Data

Table S2. Descriptive characteristics of participants with or without missing data.

|  | **No missing values (N=946)** | **Missing values (N=19)** |
| --- | --- | --- |
| **condition** |  |  |
| No Feedback (control) | 318 (33.6%) | 7 (36.8%) |
| Non-tailored Feedback | 313 (33.1%) | 6 (31.6%) |
| Tailored Feedback | 315 (33.3%) | 6 (31.6%) |
| **t0_phq9_sum** |  |  |
| M (95% CI) | 14.8 (14.5; 15.1) | 13.4 (11.9; 14.8) |
| **t0_phq9_1** |  |  |
| M (95% CI) | 1.8 (1.7; 1.8) | 1.8 (1.4; 2.2) |
| **t0_phq9_2** |  |  |
| M (95% CI) | 1.8 (1.8; 1.9) | 1.5 (1.2; 1.9) |
| **t0_phq9_3** |  |  |
| M (95% CI) | 2.1 (2.1; 2.2) | 1.9 (1.4; 2.3) |
| **t0_phq9_4** |  |  |
| M (95% CI) | 2.3 (2.3; 2.4) | 2.1 (1.7; 2.5) |
| **t0_phq9_5** |  |  |
| M (95% CI) | 1.8 (1.7; 1.8) | 1.6 (1.2; 2.0) |
| **t0_phq9_6** |  |  |
| M (95% CI) | 1.8 (1.7; 1.8) | 1.3 (0.8; 1.8) |
| **t0_phq9_7** |  |  |
| M (95% CI) | 1.7 (1.7; 1.8) | 1.7 (1.3; 2.1) |
| **t0_phq9_8** |  |  |
| M (95% CI) | 0.8 (0.7; 0.8) | 0.6 (0.2; 0.9) |
| **t0_phq9_9** |  |  |
| M (95% CI) | 0.7 (0.7; 0.8) | 0.8 (0.4; 1.3) |
| **t0_pref_1** |  |  |
| General practitioner | 327 (34.6%) | 4 (21.1%) |
| Psychotherapist | 384 (40.6%) | 12 (63.2%) |
| I don’t know | 235 (24.8%) | 3 (15.8%) |
| **t0_vordia_0** |  |  |
| Never | 560 (59.2%) | 11 (57.9%) |
| In the last year (excluded) | 0 (0%) | 0 (0%) |
| In the last five years | 199 (21.0%) | 2 (10.5%) |
| In the last ten years | 109 (11.5%) | 5 (26.3%) |
| More than ten years ago | 78 (8.2%) | 1 (5.3%) |
| **t0_nikotin_1** |  |  |
| No | 697 (73.7%) | 12 (63.2%) |
| Yes | 249 (26.3%) | 7 (36.8%) |
| **t0_alk_sum** |  |  |
| M (95% CI) | 2.7 (2.5; 2.8) | 3.2 (1.9; 4.6) |
| Missing | 0 (0%) | 2 (10.5%) |
| **t0_eq5d_1** |  |  |
| M (95% CI) | 0.6 (0.6; 0.7) | 1.0 (0.4; 1.6) |
| **t0_eq5d_2** |  |  |
| M (95% CI) | 0.3 (0.3; 0.4) | 0.4 (0.0; 0.8) |
| **t0_eq5d_3** |  |  |
| M (95% CI) | 1.0 (0.9; 1.1) | 1.3 (0.8; 1.8) |
| **t0_eq5d_4** |  |  |
| M (95% CI) | 1.4 (1.4; 1.5) | 1.6 (1.1; 2.2) |
| **t0_eq5d_5** |  |  |
| M (95% CI) | 2.1 (2.0; 2.1) | 1.6 (1.1; 2.1) |
| **t0_eq5d_6** |  |  |
| M (95% CI) | 57.6 (56.3; 59.0) | 51.0 (38.8; 63.2) |
| Missing | 0 (0%) | 3 (15.8%) |
| **t0_rfs_sum** |  |  |
| M (95% CI) | 6.0 (5.9; 6.2) | 5.4 (3.7; 7.1) |
| **severity** |  |  |
| Moderate: PHQ-9 10 – 14 | 527 (55.7%) | 13 (68.4%) |
| Severe: PHQ-9 >= 15 | 419 (44.3%) | 6 (31.6%) |
| **t0_demo_1** |  |  |
| < 4,999 residents | 159 (16.8%) | 1 (5.3%) |
| 5,000 to 19,999 residents | 159 (16.8%) | 4 (21.1%) |
| 20,000 to 99,999 residents | 137 (14.5%) | 4 (21.1%) |
| >= 100,000 residents | 491 (51.9%) | 10 (52.6%) |
| **t0_demo_3** |  |  |
| Statutory | 872 (92.2%) | 17 (89.5%) |
| Private | 74 (7.8%) | 2 (10.5%) |
| **t0_demo_9** |  |  |
| No | 844 (89.2%) | 17 (89.5%) |
| Yes | 102 (10.8%) | 2 (10.5%) |
| **t0_demo_10** |  |  |
| No | 847 (89.5%) | 16 (84.2%) |
| Yes | 99 (10.5%) | 3 (15.8%) |
| **t0_demo_11** |  |  |
| Female | 675 (71.4%) | 9 (47.4%) |
| Male | 262 (27.7%) | 10 (52.6%) |
| Diverse | 9 (1.0%) | 0 (0%) |
| **t0_demo_12** |  |  |
| M (95% CI) | 37.2 (36.3; 38.1) | 47.2 (40.3; 54.0) |
| **t0_demo_13** |  |  |
| Married/in a relationship | 445 (47.0%) | 12 (63.2%) |
| Single, divorced, widowed, other | 501 (53.0%) | 7 (36.8%) |
| **t0_demo_14** |  |  |
| Living together with others | 633 (66.9%) | 13 (68.4%) |
| Living alone | 313 (33.1%) | 6 (31.6%) |
| **t0_demo_17** |  |  |
| Not working | 132 (14.0%) | 2 (10.5%) |
| Retired | 77 (8.1%) | 4 (21.1%) |
| Unemployed | 47 (5.0%) | 0 (0%) |
| Working part-time or by the hour | 261 (27.6%) | 4 (21.1%) |
| Employed | 429 (45.3%) | 8 (42.1%) |
| Missing | 0 (0%) | 1 (5.3%) |
| **t0_demo_15_mod** |  |  |
| Low (< 10 years of education) | 161 (17.0%) | 7 (36.8%) |
| Middle (>= 10 years of education) | 297 (31.4%) | 5 (26.3%) |
| High (university entrance qualification) | 488 (51.6%) | 7 (36.8%) |
| **t0_bipq_9** |  |  |
| No | 133 (14.1%) | 3 (15.8%) |
| Maybe | 428 (45.2%) | 9 (47.4%) |
| Yes | 385 (40.7%) | 7 (36.8%) |
| **t0_bipq_1** |  |  |
| M (95% CI) | 6.4 (6.3; 6.6) | 5.9 (4.9; 7.0) |
| **t0_bipq_2** |  |  |
| M (95% CI) | 6.7 (6.6; 6.9) | 6.3 (4.7; 7.8) |
| **t0_bipq_3** |  |  |
| M (95% CI) | 4.8 (4.7; 5.0) | 5.0 (3.9; 6.1) |
| **t0_bipq_4** |  |  |
| M (95% CI) | 6.2 (6.0; 6.3) | 5.9 (4.7; 7.2) |
| **t0_bipq_5** |  |  |
| M (95% CI) | 6.6 (6.4; 6.7) | 5.7 (4.3; 7.1) |
| **t0_bipq_6** |  |  |
| M (95% CI) | 6.5 (6.4; 6.7) | 6.2 (4.9; 7.4) |
| **t0_bipq_7** |  |  |
| M (95% CI) | 7.4 (7.3; 7.5) | 6.0 (4.7; 7.3) |
| **t0_bipq_8** |  |  |
| No, I know these complaints from earlier phases of my life | 747 (79.0%) | 15 (78.9%) |
| Yes, this is the first time | 199 (21.0%) | 3 (15.8%) |
| Missing | 0 (0%) | 1 (5.3%) |
| **t0_sss8_1** |  |  |
| M (95% CI) | 1.6 (1.5; 1.6) | 1.3 (0.7; 2.0) |
| **t0_sss8_2** |  |  |
| M (95% CI) | 1.9 (1.8; 2.0) | 1.9 (1.3; 2.5) |
| **t0_sss8_3** |  |  |
| M (95% CI) | 1.5 (1.5; 1.6) | 1.6 (1.0; 2.2) |
| **t0_sss8_4** |  |  |
| M (95% CI) | 1.7 (1.6; 1.8) | 1.1 (0.6; 1.7) |
| **t0_sss8_5** |  |  |
| M (95% CI) | 1.2 (1.1; 1.3) | 0.9 (0.4; 1.4) |
| **t0_sss8_6** |  |  |
| M (95% CI) | 1.0 (1.0; 1.1) | 1.2 (0.6; 1.8) |
| **t0_sss8_7** |  |  |
| M (95% CI) | 3.1 (3.0; 3.2) | 2.8 (2.2; 3.5) |
| **t0_sss8_8** |  |  |
| M (95% CI) | 2.5 (2.4; 2.6) | 2.4 (1.8; 3.1) |
| **t0_sss8_sum** |  |  |
| M (95% CI) | 14.5 (14.1; 14.8) | 13.3 (10.4; 16.1) |
| **t0_gad7_1** |  |  |
| M (95% CI) | 2.0 (1.9; 2.0) | 1.4 (1.1; 1.8) |
| **t0_gad7_2** |  |  |
| M (95% CI) | 1.8 (1.7; 1.8) | 1.2 (0.9; 1.4) |
| **t0_gad7_3** |  |  |
| M (95% CI) | 1.9 (1.8; 2.0) | 1.3 (0.9; 1.7) |
| **t0_gad7_4** |  |  |
| M (95% CI) | 2.2 (2.1; 2.2) | 1.9 (1.5; 2.3) |
| **t0_gad7_5** |  |  |
| M (95% CI) | 1.3 (1.3; 1.4) | 1.2 (0.8; 1.6) |
| **t0_gad7_6** |  |  |
| M (95% CI) | 1.7 (1.6; 1.7) | 1.6 (1.3; 2.0) |
| **t0_gad7_7** |  |  |
| M (95% CI) | 1.4 (1.4; 1.5) | 1.2 (0.7; 1.6) |
| Missing | 0 (0%) | 1 (5.3%) |
| **t0_gad7_sum** |  |  |
| M (95% CI) | 12.2 (11.9; 12.5) | 9.6 (7.6; 11.6) |
| Missing | 0 (0%) | 1 (5.3%) |
| **t0_deprisk_1** |  |  |
| No | 267 (28.2%) | 10 (52.6%) |
| Yes | 679 (71.8%) | 9 (47.4%) |
| **t0_deprisk_2** |  |  |
| No | 811 (85.7%) | 14 (73.7%) |
| Yes | 135 (14.3%) | 3 (15.8%) |
| Missing | 0 (0%) | 2 (10.5%) |
| **t0_deprisk_3** |  |  |
| No | 240 (25.4%) | 7 (36.8%) |
| Yes | 706 (74.6%) | 12 (63.2%) |
| **t0_deprisk_4** |  |  |
| No | 417 (44.1%) | 9 (47.4%) |
| Yes | 529 (55.9%) | 9 (47.4%) |
| Missing | 0 (0%) | 1 (5.3%) |
| **t0_deprisk_5** |  |  |
| No | 58 (6.1%) | 4 (21.1%) |
| Yes | 888 (93.9%) | 14 (73.7%) |
| Missing | 0 (0%) | 1 (5.3%) |
| **t0_deprisk_6** |  |  |
| No | 640 (67.7%) | 10 (52.6%) |
| Yes | 306 (32.3%) | 8 (42.1%) |
| Missing | 0 (0%) | 1 (5.3%) |
| **t0_deprisk_8** |  |  |
| No | 367 (38.8%) | 2 (10.5%) |
| Yes | 579 (61.2%) | 15 (78.9%) |
| Missing | 0 (0%) | 2 (10.5%) |
| **t0_deprisk_9** |  |  |
| Never | 606 (64.1%) | 10 (52.6%) |
| In the last year | 44 (4.7%) | 2 (10.5%) |
| In the last five years | 138 (14.6%) | 1 (5.3%) |
| In the last ten years | 81 (8.6%) | 3 (15.8%) |
| More than ten years ago | 77 (8.1%) | 3 (15.8%) |
| **t0_deprisk_10** |  |  |
| No | 373 (39.4%) | 7 (36.8%) |
| Yes | 573 (60.6%) | 10 (52.6%) |
| Missing | 0 (0%) | 2 (10.5%) |
| **t0_deprisk_11** |  |  |
| No | 696 (73.6%) | 11 (57.9%) |
| Yes | 250 (26.4%) | 3 (15.8%) |
| Missing | 0 (0%) | 5 (26.3%) |

### Comparison of no feedback with tailored feedback

Table S3. Best linear projection for the top four predictors of the causal forest based on training data comparing no feedback with tailored feedback.

| **Term** | **Estimate** | **SE** | ***P value*** |
| --- | --- | --- | --- |
| **How old are you? (years)** | -0.01 | 0.04 | .76 |
| **SSS-8 Sum score (0 to 32)** | 0.16 | 0.12 | .18 |
| **GAD-7 Sum score (0 to 21)** | -0.05 | 0.16 | .74 |
| **Illness Perception Item 4:** How much do you think a treatment can help with these complaints? (0 not at all to 10 extremely helpful) | 0.3 | 0.21 | .14 |

*Note.* GAD-7 = Generalized Anxiety Disorder Scale-7, SSS-8 = Somatic Symptom Scale-8.


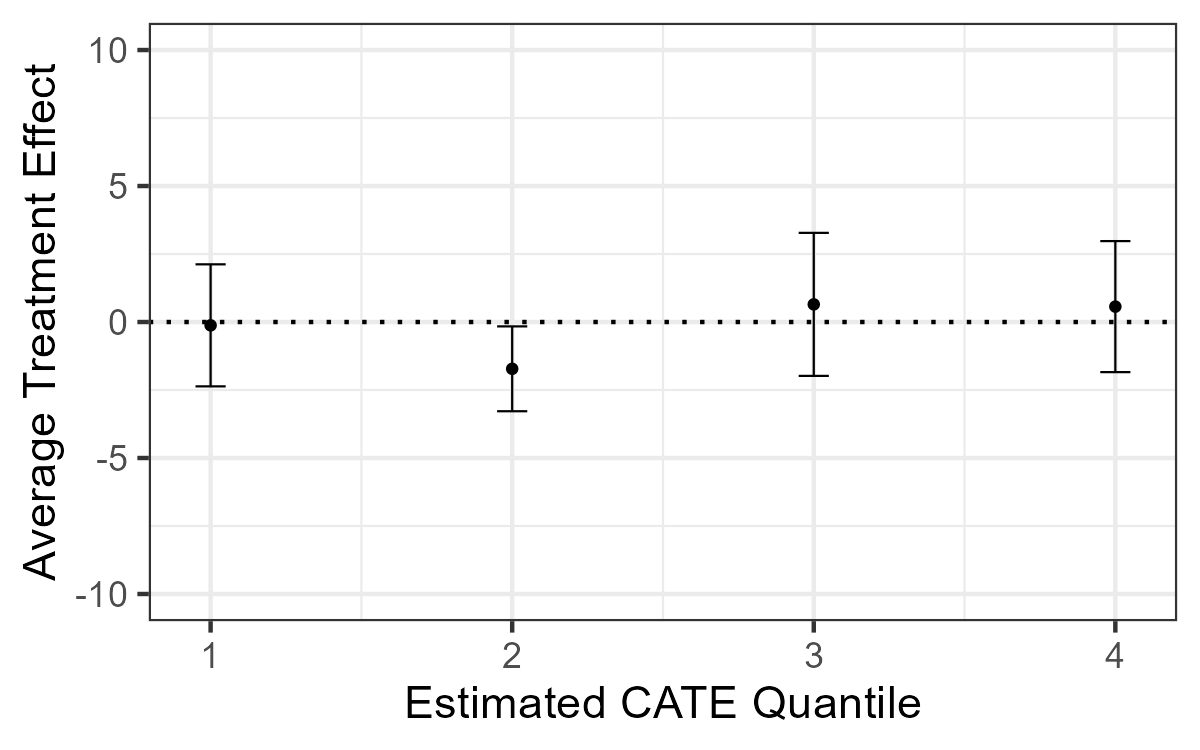


Figure S1. Doubly robust average treatment effects in participant groups reflecting quantiles of predicted CATE from lowest (l) to highest (r). CATE was predicted in test data with the tau-forest. ATE was estimated within quantiles with the evaluation forest (based on test data). Positive values indicate less favorable ATE.


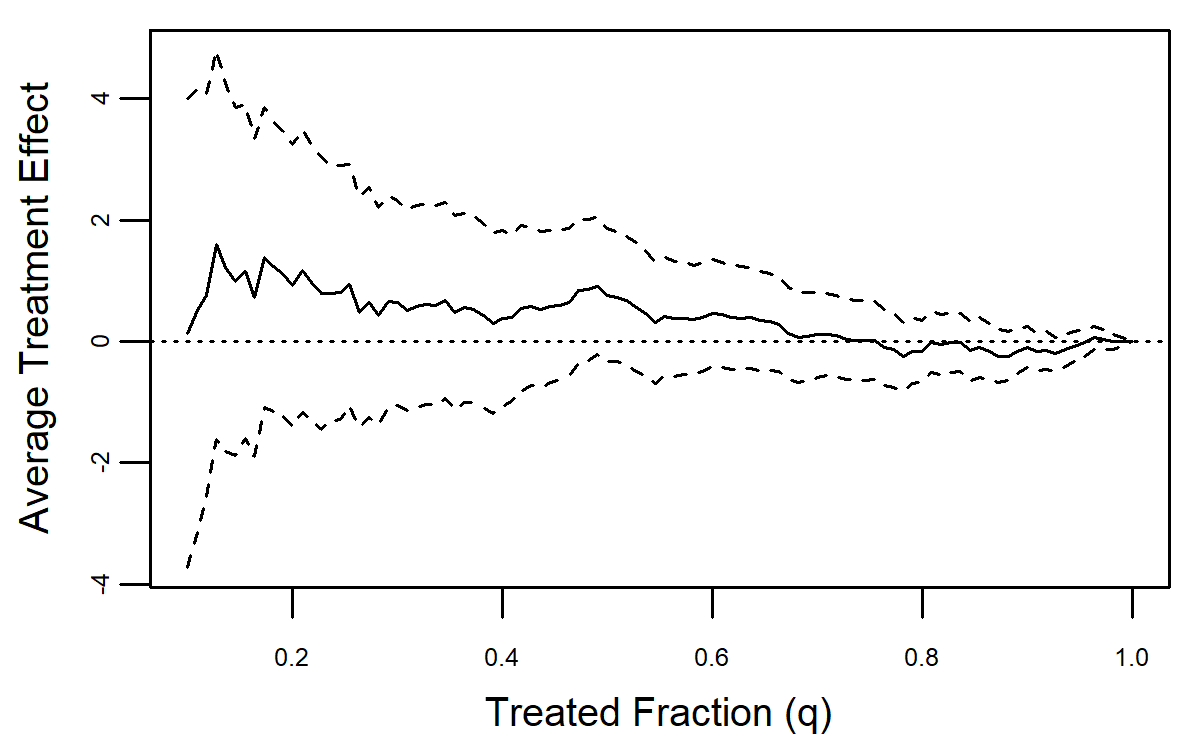


Figure S2. Targeting operator characteristic curve plot. The dashed lines are pointwise 95% confidence intervals conditional on the estimated CATE function, i.e., the tau-forest based on the training data. The y-lab illustrates the benefit of providing feedback only to a fraction of participants based on their CATE (i..e, treatment priority score), over treating everyone (difference in average treatment effects; i.e., PHQ-9 change six months after screening). The x lab illustrates the fraction treated from highest (left) to lowest (right) CATE. Positive values indicate less favorable ATE.


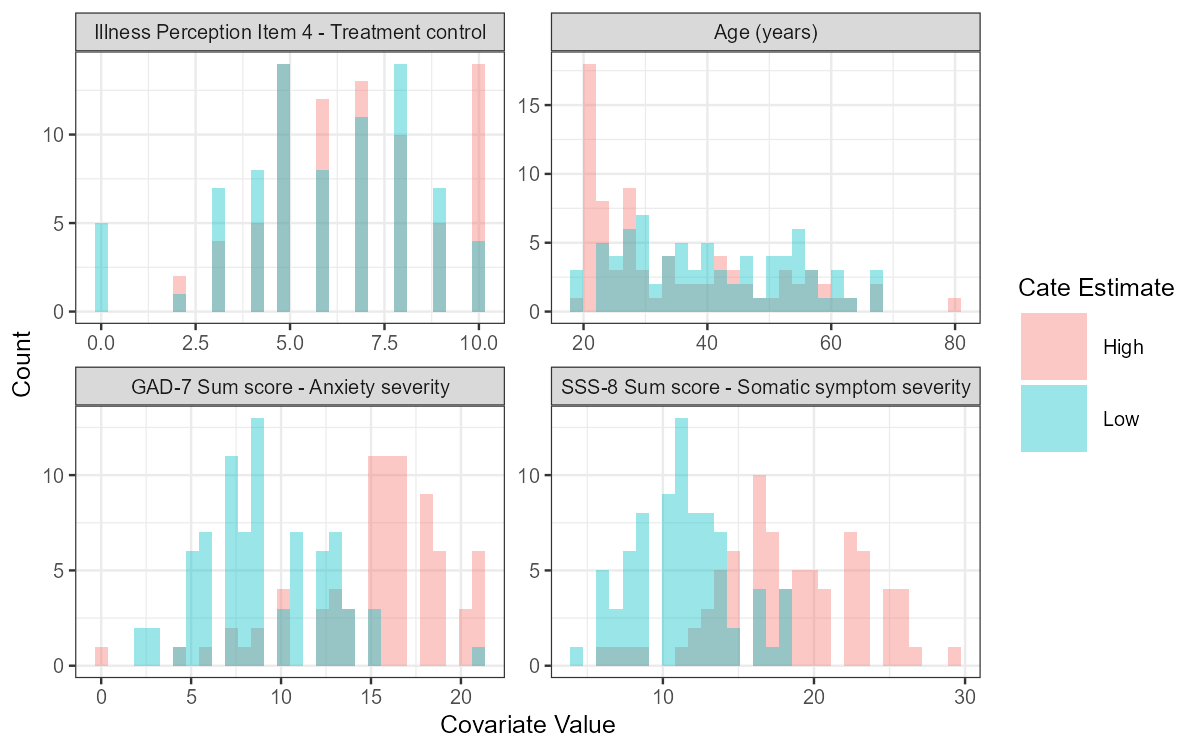


Figure S3. Covariate profiles for participants with high (upper 25%, magenta) or low (lower 25%, cyan) CATE predicted in test data with the tau-forest. Illustrated are the top four most important variables of the tau-forest.

### Comparison of no feedback with any feedback

Table S4. Best linear projection for the top four predictors of the causal forest based on training data comparing no feedback with any feedback.

| **Term** | **Estimate** | **SE** | ***P*** |
| --- | --- | --- | --- |
| **Illness Perception Item 4:** How much do you think a treatment can help with these complaints? (0 not at all to 10 extremely helpful) | 0.37 | 0.19 | .06 |
| **Illness Perception Item 3:** How much control do you feel you have over your complaints? (0 no control at all to 10 full control) | 0.58 | 0.23 | .01 |
| **Illness Perception Item 5:** How concerned are you about your complaints? (0 not concerned at all to 10 extremely concerned) | 0.23 | 0.26 | .38 |
| **Illness Perception Item 7:** How much do these complaints affect you emotionally? (0 not at all to 10 extremely) | 0.24 | 0.3 | .42 |

*Note.* GAD-7 = Generalized Anxiety Disorder Scale-7, SSS-8 = Somatic Symptom Scale-8.


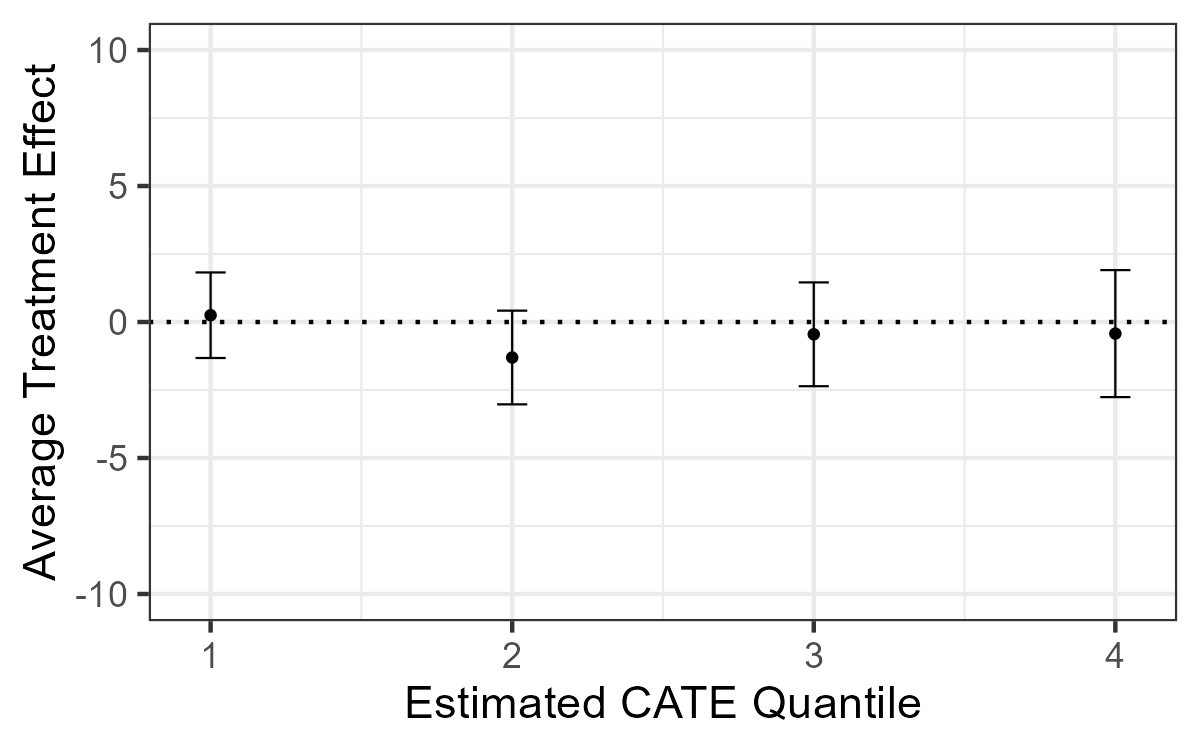


Figure S4. Doubly robust average treatment effects in participant groups reflecting quantiles of predicted CATE from lowest (l) to highest (r). CATE was predicted in test data with the tau-forest. ATE was estimated within quantiles with the evaluation forest (based on test data). Positive values indicate less favorable ATE.


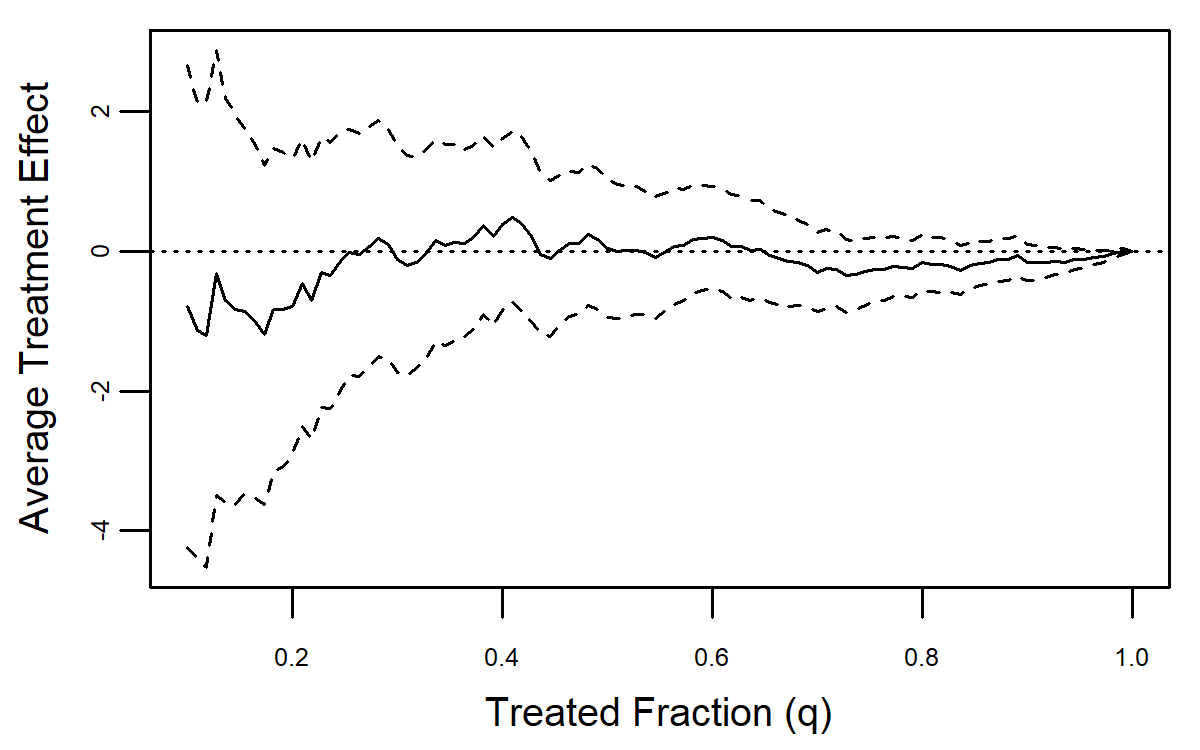


Figure S5. Targeting operator characteristic curve plot. The dashed lines are pointwise 95% confidence intervals conditional on the estimated CATE function, i.e., the tau-forest based on the training data. The y-lab illustrates the benefit of providing feedback only to a fraction of participants based on their CATE (i..e, treatment priority score), over treating everyone (difference in average treatment effects; i.e., PHQ-9 change six months after screening). The x lab illustrates the fraction treated from highest (left) to lowest (right) CATE. Positive values indicate less favorable ATE.


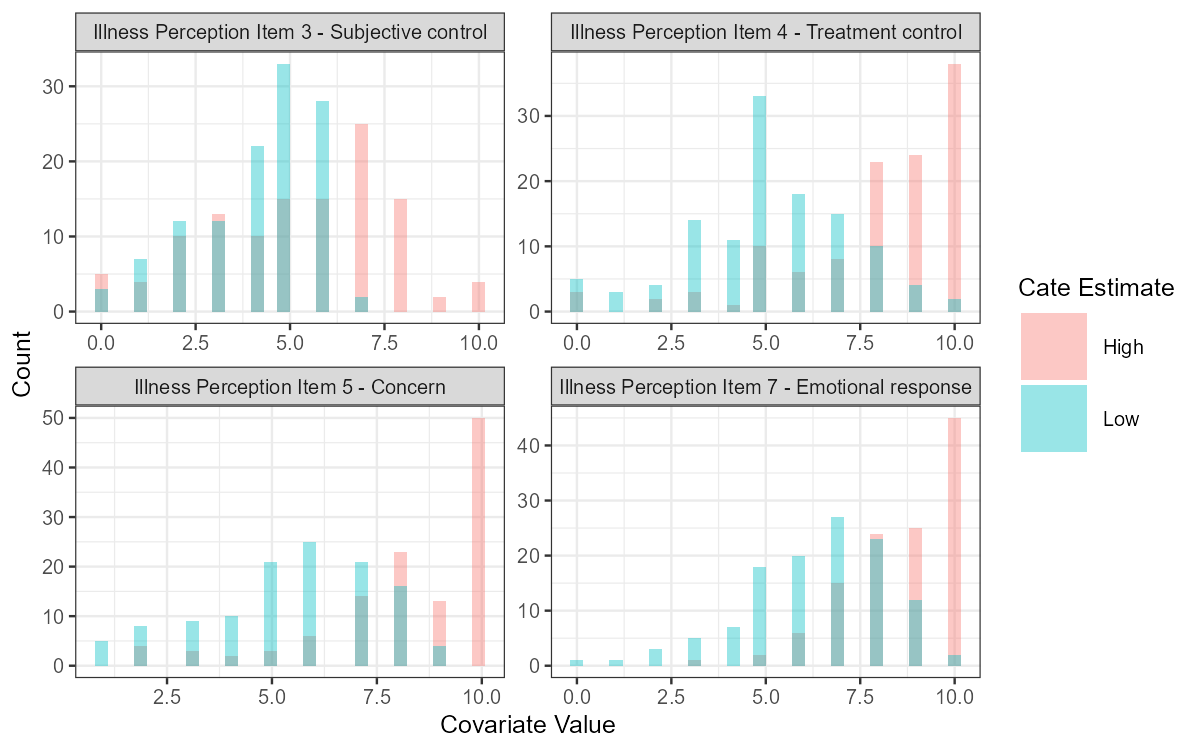


Figure S6. Covariate profiles for participants with high (upper 25%, magenta) or low (lower 25%, cyan) CATE predicted in test data with the tau-forest. Illustrated are the top four most important variables of the tau-forest.

### Calibration

Table S5. Testing presence of heterogeneity in treatment effect via calibration.

| **Comparison** | **Calibration (based on training data)** | |
| --- | --- | --- |
|  | **Estimate** | ***P value*** |
|  |  |  |
| **No Feedback vs. Non-tailored Feedback** |  |  |
| Mean forest prediction^a^ | 1.97 | .47 |
| Differential forest prediction^b^ | 1.49 | .09 |
|  |  |  |
| **No Feedback vs. Tailored Feedback** |  |  |
| Mean forest prediction^a^ | 0.93 | .25 |
| Differential forest prediction^b^ | -8.05 | .99 |
|  |  |  |
| **No Feedback vs. Any Feedback** |  |  |
| Mean forest prediction^a^ | 1.32 | .43 |
| Differential forest prediction^b^ | -2.01 | .87 |

^a^Mean forest prediction estimates closer to 1 indicate correct mean prediction.

^b^Differential forest prediction reflects an omnibus test for HTE, with a coefficient of 1 indicating perfect calibration of heterogeneity estimates. P-value < .05 would indicate presence of HTE.

Box S1. Using causal forests to estimate heterogeneity of treatment effects.

Random forests (RF) iteratively partition individuals based on impurity-reduction criteria on a random subset of covariates into neighborhoods. Random selection of covariates allows the creation of a forest of distinct decision trees [7]. To predict an individual’s outcome, each subject flows through every decision tree. Mean outcomes in determined neighborhoods are then averaged across all trees in the forest.

CF are a generalized form of RF and can be used to estimate CATE. As such, CF partition the data to maximize heterogeneity in treatment effect estimates (i.e., CATE) between participants with distinct covariate profiles [6, 8].

CF run weighted residual-on-residual regressions within subgroups with similar covariate profiles, with weights capturing similarity in covariates [17].

In contrast to regular RF CF introduce two restrictions honesty and subsampling to establish independence of model training and effect estimation [6, 8]. Honesty is established by using each individual either for CATE estimation, or for the definition of splitting rules during model training [6]. Subsampling, refers to choosing observations without replacement.

Further details on the causal forest algorithm are available online [https://grf-labs.github.io/grf/articles/grf_guide.html].

Box S2. Model evaluation of causal forests.

To evaluate CATE functions, we ran several sensitivity analyses following previous work of Sverdrup, Petukhova [4]. These procedures invoke training of two CF. A first CF is based on the training data only (tau-forest, random split 1:1). A second CF is based on unseen test data (eval-forest), allowing later evaluation of the CATE function under different hypothetical treatment allocation procedures (i.e., treatment prioritization) which provides a doubly robust test of presence of HTE.

Evaluation strategy:

- First, the average difference in potential outcomes is estimated via ATE, based on the tau-forest.
- Second, rank average treatment effects are utilized to plot the targeting operator characteristic (TOC) curve, which allows an evaluation of ATE under different treatment prioritization rules. More precisely, the TOC-curve depicts the difference in ATEs (determined in eval-forest) when treatment allocation is random versus when treatment allocation is based on the magnitude of predicted CATE (predicted with tau-forest) [4, 5]. Point estimates are derived for every *q^th^* quantile of CATE predictions, ordered by magnitude from highest to lowest $(1)$ [4].

|  | $TOC\left( q \right)=E\left[ Y_{i}\left( 1 \right)-Y_{i}\left( 0 \right) \right\vert Estimated CATE\left( X_{i} \right)\geq1-q]-ATE$ | $(1)$ |
| --- | --- | --- |

- Third, we estimate the Rank-Weighted Average Treatment Effect via computation of the area under the TOC curve (AUTOC) with bootstrapped standard errors (r = 200 bootstrap replicates). In order to test significance of the estimated CATE-function and thus presence of HTE, we follow previously published recommendations and compute a *t*-value based on AUTOC and its variation (2). Given a standard random normal, significance is determined two-sided with a type I error rate of α = 5% [4].

|  | $t=\hat{AUTOC}/\sqrt{Var(\hat{AUTOC})}$ | $(2)$ |
| --- | --- | --- |

- Fourth, we identify the top four most important predictors of the tau forest, with variable importance denoting the number of times a predictor is used to partition our data across trees regarding symptom severity change given treatment.
- Fifth, best linear projections (BLP) were computed to examine average effects of the top four predictors regarding the CATE function. BLP is the optimal prediction of CATE based on a predictor *Zi* (3) [4].

|  | $\tau(Xi) \sim\alpha+ \beta Zi + \varepsilon$ | $(3)$ |
| --- | --- | --- |

- Sixth we illustrate covariate profiles based on the highest and lowest quartile of predicted CATEs for the top four predictors.
- Seventh, we test calibration of trained models.
